# The Long-Term Prognostic Significance of 6-Minute Walk Test Distance in Patients with Chronic Heart Failure

**DOI:** 10.1155/2014/505969

**Published:** 2014-03-27

**Authors:** Lee Ingle, John G. Cleland, Andrew L. Clark

**Affiliations:** ^1^Department of Sport, Health & Exercise Science, University of Hull, 104 Don Building, Cottingham Road, Kingston upon Hull, HU7 6RX, UK; ^2^National Heart & Lung Institute, Royal Brompton & Harefield Hospitals, Imperial College, London, UK; ^3^Department of Cardiology, Castle Hill Hospital, Kingston uponHull, HU16 5JQ, UK

## Abstract

*Background*. The 6-minute walk test (6-MWT) is used to assess patients with chronic heart failure (CHF). The prognostic significance of the 6-MWT distance during long-term followup (>5 years) is unclear. *Methods*. 1,667 patients (median [inter-quartile range, IQR]) (age 72 [65–77]; 75% males) with heart failure due to left ventricular systolic impairment undertook a 6-MWT as part of their baseline assessment and were followed up for 5 years. *Results*. At 5 years' followup, those patients who died (*n* = 959) were older at baseline and had a higher log NT pro-BNP than those who survived to 5 years (*n* = 708). 6-MWT distance was lower in those who died [163 (153) m versus 269 (160) m; *P* < 0.0001]. Median 6-MWT distance was 300 (150–376) m, and quartile ranges were <46 m, 46–240 m, 241–360 m, and >360 m. 6-MWT distance was a predictor of all-cause mortality (HR 0.97; 95% CI 0.96-0.97; Chi-square = 184.1; *P* < 0.0001). Independent predictors of all-cause mortality were decreasing 6-MWT distance, increasing age, increasing NYHA classification, increasing log NT pro-BNP, decreasing diastolic blood pressure, decreasing sodium, and increasing urea. *Conclusion*. The 6-MWT is an important independent predictor of all-cause mortality following long-term followup in patients with CHF.

## 1. Introduction

Functional capacity is strongly related to survival in patients with chronic heart failure (CHF) [1]. Although cardiopulmonary exercise testing (CPET) with metabolic gas exchange measurements is perhaps the “gold standard” method for assessing exercise capacity, it is not widely available, and so more simple tests are commonly used [2]. The 6-minute walk test (6-MWT) is reproducible and sensitive to changes in quality of life [3–5]. It is a self-paced, submaximal test, and exercise intensity mimics activities of daily living in patients with mild-to-moderate heart failure [6–9]. Thus, the 6-MWT may suit patients with CHF who may experience symptoms such as breathlessness below their peak exercise capacity.

We have previously reported that decreasing 6-MWT distance was an independent predictor of increasing mortality in patients with left ventricular systolic dysfunction but that it was a less potent single predictor than N-terminal pro-brain natriuretic peptide (NT pro-BNP) [10]. Others have shown that the 6-MWT distance is a less powerful predictor of outcome than some variables, such as VE/VCO_2_ slope and peak oxygen uptake derived from CPET [11].

The aim of the present study was to assess the long-term (>5 years) prognostic significance of the 6-MWT distance in a large sample of patients with CHF.

## 2. Methods

The Hull and East Riding Ethics Committee approved the study, and all patients provided informed consent for participation. Clinical information obtained included past medical history and drug and smoking history. Clinical examination included assessment of body mass index (BMI), heart rate, rhythm, and blood pressure (BP). Heart failure was defined as current symptoms of heart failure, or a history of symptoms controlled by ongoing therapy, in the presence of reduced left ventricular (LV) systolic function on echocardiography and in the absence of any other cause for symptoms [12, 13]. 2D echocardiography was carried out by one of three trained operators. LV function was assessed by estimation on a scale of normal, mild, mild-to-moderate, moderate, moderate-to-severe, and severe impairment. LV ejection fraction (LVEF) was calculated using Simpson's formula, where possible, from measurements of end-diastolic and end-systolic volumes on apical 2D views, following the guidelines of Schiller et al. [14] and LVSD was diagnosed if LVEF was <45%.

The 6-MWT was conducted following a standardised protocol [9, 10]. A 15 m flat, obstacle-free corridor, with chairs placed at either end, was used. Patients were instructed to walk as far as possible, turning 180° every 15 m in the allotted time of 6 min. Patients were able to rest, if needed, and the time remaining was called every second minute [15]. Patients were excluded if they were unable to walk without assistance from another person (not including mobility aids), or if they were unable to exercise because of noncardiac limitations. Patients walked unaccompanied so as not to influence walking speed. After 6 min, patients were instructed to stop and the total distance covered was measured to the nearest metre. Standardised verbal encouragement was given to patients after 2 min and 4 min. If a patient could not undertake the 6-MWT, a distance of 0 m was recorded.

### 2.1. Statistical Analysis

Continuous variables are presented as median with interquartile range (IQR) or standard deviation (SD) and categorical data as percentages. Continuous variables were assessed for normality by the Kolmogorov-Smirnov test. NT pro-BNP was normalised by log-transformation for analysis. Differences between those who survived to five years and those who did not were determined by the independent samples* t*-test or Pearson's Chi-square test. No survivor was followed up for less than 5 years. We used receiver operating characteristic (ROC) curves to assess the predictive power of variables and report the area under the curve (AUC) with 95% confidence intervals (CI), sensitivity, specificity, and optimal cut-points. To define the optimal cut-point, we used the point closest to the upper left corner of the ROC curve, often known as the (0, 1) criterion.

We used Kaplan-Meier curves to display mortality data using the guidance of Pocock et al. [16]. For illustration, 6-MWT distance data were divided into quartiles (≤45 m, 46–240 m, 241–360 m, and >360 m). Cox regression models (univariable and multivariable) were used to develop predictor models using all baseline variables. We used multivariable Cox proportional hazards model using the backward likelihood ratio method (*P *value for entry was* <*0.05;* P *value for removal was* >*0.1) to identify independent predictors of all-cause mortality from candidate predictor variables. The assumption of proportionality was tested for each variable using the method of Grambsch and Therneau [17].

To minimise the risk of “overfitting,” we were guided by Peduzzi and colleagues [18, 19] who suggested an events per variable ratio of 10 : 1. To determine the robustness of our model(s), we performed bootstrapping based on 1,000 stratified samples. We checked for colinearity by calculating Pearson correlation coefficients. We used a cut-off value of 0.3 to identify colinearity. SPSS version 19.0 (IBM, New York, USA) was used to analyse the data. An arbitrary level of 5% statistical significance was used throughout (two-tailed). We followed the guidance of Perneger [20] and did not adjust for multiple testing in order to avoid the inflation of type I error. The primary outcome measure was all-cause mortality.

## 3. Results

1,667 patients (median (interquartile range, IQR)) (age 72 (65–77); 75% males) with heart failure due to left ventricular systolic impairment were included in the study. At 5-year followup, those patients who died (*n* = 959) were, at baseline, older and had a lower BMI, higher NYHA class, lower LVEF, higher creatinine, higher log NT pro-BNP, lower haemoglobin, and higher urea levels than those who survived to 5 years (*n* = 708; Table 1). 6-MWT distance was lower in those who died (163 (153) m versus 269 (160) m; *P* < 0.0001). Median 6-MWT distance was 300 (150–376) m, and quartile ranges for 6-MWT distance were <46 m, 46–240 m, 241–360 m, and >360 m.  Table 2 shows clinical characteristics divided by quartiles of 6-MWT distance. There were significant between-group differences for age, BMI, LVEF, resting HR, resting systolic/diastolic BP, QRS duration, haemoglobin, log NT pro-BNP, urea, and creatinine (all *P* < 0.05).

**Figure 1:**
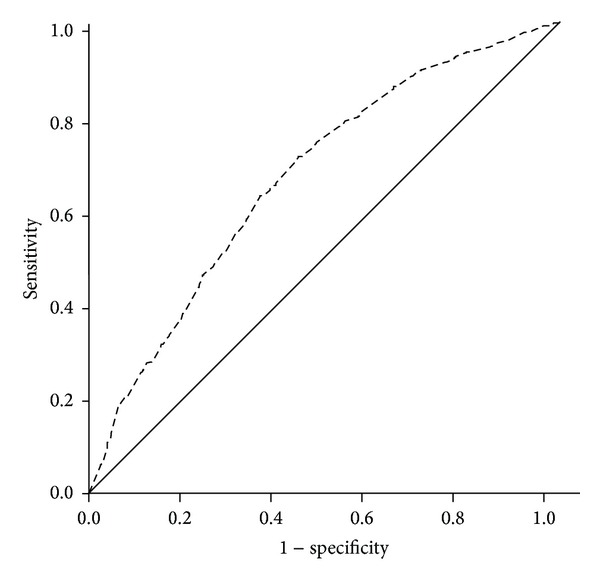
Receiver operating characteristic curve showing value of the 6-MWT for predicting all-cause mortality at 5 years in patients with CHF (AUC = 0.67; *P* < 0.0001; 95% CI = 0.64–0.70; sensitivity = 0.81; specificity = 0.57; optimal cut-point = 350 m).

**Figure 2:**
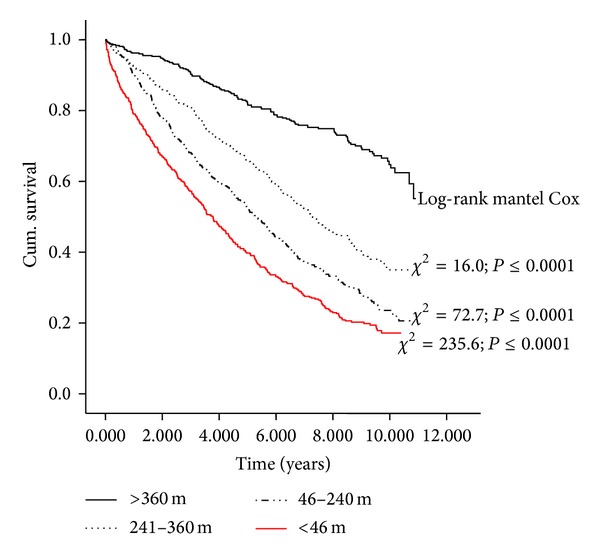
Kaplan-Meier survival curve showing quartiles of 6-MWT distance (m) (<46 m, event free survival 24%; 46–240 m, event free survival 29%; 241–360 m, event free survival 45%; >360 m, event free survival 70%).

Thirteen variables were significantly associated with all-cause mortality in univariable Cox analysis (Table 3). After bootstrapping, only 6 variables (6-MWT, age, NT pro-BNP, NYHA class, diastolic BP, and haemoglobin) remained statistically significant (Table 4). All variables in Table 1 were included in a final multivariable Cox model, and six were independent predictors of all-cause mortality, decreasing 6-MWT distance, increasing age, increasing NYHA classification, increasing NT pro-BNP, decreasing diastolic blood pressure, decreasing sodium, and increasing urea (Table 5). ROC curve analysis of 6-MWT distance and all-cause mortality at 5 years is shown in Figure 1 (AUC = 0.67; *P* < 0.0001; 95% CI = 0.64–0.70; the optimal cut-point for 6-MWT distance was 350 m with sensitivity 0.81 and specificity 0.57).  Figure 2 shows a Kaplan-Meier survival curve for the patients divided by quartiles of 6-MWT distance (<46 m: event free survival 24%; 46–240 m: event free survival 29%; 241–360 m: event free survival 45%; >360 m: event free survival 70%).

## 4. Discussion

We have shown that the 6-MWT is an independent predictor of all-cause mortality during long-term (5 year) followup in patients with CHF. To our knowledge, this is the largest study that has focused on the prognostic value of 6-MWT distance during extended followup. We have previously shown that 6-MWT distance is an independent predictor of risk following medium-term followup (median 36.6 (28–45) months). In 1,592 patients, 212 died representing a crude death rate of 13.3%. Five independent predictors of all-cause mortality were identified including decreasing 6-MWT distance [10].

Other large-scale studies including the SENIORS trial [21] (*n* = 2, 128 patients, ≥70 years with LVEF ≤ 35% or recent hospital admission) have also shown that 6-MWT distance is an independent predictor of mortality over a modest time period (mean followup: 21 months) [21]. Another study [22] (mean followup 34 months) has also confirmed the prognostic value of 6-MWT distance for predicting cardiac-related death in patients with mild-to-moderate CHF. Long-term studies have been reported in patients with stable coronary heart disease, including the Heart and Soul Study [23] which followed up patients for a median of 8.0 (4.2–9.0) years and showed that 6-MWT distance predicted cardiovascular events and provided similar prognostic value to treadmill exercise capacity. A limitation of the study was a small sample size (*n* = 556) and a limited number of events (184 deaths).

A number of studies have shown that 6-MWT distance is a less powerful predictor of outcome in patients with CHF than variables derived from CPET such as VE/VCO_2_ slope and peak oxygen uptake [24]. Opasich and colleagues [25] concluded that 6-MWT distance (mean followup 387 ± 177 days) does not provide complimentary prognostic information or should be substituted for peak oxygen consumption. In a study of only 253 patients with either systolic or diastolic heart failure in whom there were 43 cardiac events over 4 years, Guazzi et al. [11] found that although 6-MWT distance correlated with peak oxygen uptake and VE/VCO_2_ slope, there was no significant association between 6-MWT distance and survival. However, CPET-derived variables were predictors of prognosis.

Most studies using CPET variables as potential predictors of outcome have followed up patients for two years or less. Studies have reported very short-term (e.g., 6 months or less) followup [26, 27], 12 months [28–31] or up to 2 years [26, 32–37]. Few studies have reported tracking periods beyond 3 years [38, 39]. Prognostic models for patients with heart failure usually contain variables from domains measuring some aspect of exercise capacity, some indicator of cardiac function (such as left ventricular ejection fraction), and some indicator of systemic involvement (such as creatinine). CPET is not widely available. We show here that the simple and cheap 6-MWT distance is an easily obtainable variable which strongly relates to long-term survival in patients with CHF.

### 4.1. Study Limitations

The 6-MWT is not a test of maximal exercise capacity but is a test of submaximal exercise performance [6]. The American Thoracic Society [7] advocates that verbal encouragement should be limited and tone of voice be controlled during the 6-MWT in an elderly, chronic disease population. We have followed this approach with our patients but different centres will operate different systems. Therefore, findings from our current study should not be extrapolated to other populations or to other research centres that may use a more aggressive 6-MWT coaching style.

### 4.2. Conclusion

The 6-MWT is an independent predictor of all-cause mortality following long-term (5-year) followup in patients with CHF. It provides similar or better discriminatory power than other routinely collected physical and biochemical variables and, as such, might make a reasonable target for treatment.

## Figures and Tables

**Table 1:** Baseline characteristics of patients [mean (SD)] divided by survival to >5 years.

Variables	Dead (*n* = 959)	Alive (*n* = 708)	*P* value
Age (years)	74.1 (8.9)	67.9 (10.6)	**<0.0001**
Males (%)	73	75	0.184
BMI (kg·m^−2^)	27.7 (5.7)	28.9 (5.6)	**<0.0001**
NYHA class			**<0.0001**
I/II	36	64	
III/IV	58	42	
LVEF (%)	33 (10)	36 (9)	**<0.0001**
FEV_1_/FVC (%)	66 (16)	70 (16)	**<0.0001**
Resting HR (beats·min^−1^)	76 (18)	74 (20)	0.165
Resting systolic BP (mmHg)	132 (26)	133 (24)	0.158
Resting diastolic BP (mmHg)	74 (14)	78 (14)	**<0.0001**
QRS duration (ms)	121 (32)	112 (29)	**<0.0001**
Haemoglobin (g·dL^−1^)	13.0 (1.7)	13.9 (1.5)	**<0.0001**
Log NT pro-BNP*	7.7 (1.2)	6.7 (1.3)	**<0.0001**
Sodium (mmol·L^−1^)	139 (4)	139 (3)	0.652
Potassium (mmol·L^−1^)	4.4 (0.5)	4.4 (0.5)	0.777
Urea (mmol·L^−1^)	9.3 (4.7)	7.1 (3.6)	**<0.0001**
Creatinine (u·moL^−1^)	130 (51)	110 (55)	**<0.0001**
Diuretic (%)	83	84	0.760
ACE-inhibitor (%)	78	77	0.348
Beta-blocker (%)	73	69	0.322
Spironolactone (%)	22	20	0.202
6-MWT (m)	163 (153)	269 (160)	**<0.0001**

NYHA: New York Heart Association; BMI: body mass index; LVI: left ventricular impairment; LVEF*: left ventricular ejection fraction available in 67% of patients; 6-MWT: 6-min walk test; BP: blood pressure; log NT pro-BNP (pg·mL^−1^)* available in 92% of patients.

**Table 2:** Clinical characteristics separated by quartiles of 6-MWT distance in patients with CHF (mean ± SD).

Variables	≤45 m	46–240 m	241–360 m	>360 m	*P* value
Age (years)	72.4 (10.6)	72.9 (9.6)	71.3 (8.8)	64.9 (10.6)	**<0.0001**
BMI (kg·m^−2^)	27.5 (4.5)	27.7 (5.2)	28.5 (5.8)	28.9 (6.8)	**0.002**
LVEF (%)	32.0 (10.0)	35.1 (10.7)	34.6 (9.2)	36.2 (9.3)	**<0.0001**
FEV_1_/FVC (%)	66.4 (17.7)	67.5 (15.0)	67.3 (16.5)	68.8 (16.4)	0.257
Resting HR (beats·min^−1^)	80 (36)	75 (17)	73 (17)	72 (17)	**<0.0001**
Resting systolic BP (mmHg)	129 (29)	134 (25)	136 (23)	135 (23)	**<0.0001**
Resting diastolic BP (mmHg)	74 (14)	77 (15)	77 (14)	80 (14)	**<0.0001**
QRS duration (ms)	117 (31)	119 (33)	120 (32)	114 (30)	**0.045**
Haemoglobin (g·dL^−1^)	13.0 (1.9)	13.2 (1.7)	13.6 (1.5)	14.1 (1.4)	**<0.0001**
Log NT pro-BNP	7.5 (1.4)	7.3 (1.4)	7.0 (1.2)	6.5 (1.3)	**<0.0001**
Sodium (mmol·L^−1^)	138 (3.9)	139 (3.8)	139 (3.3)	139 (3.0)	0.241
Potassium (mmol·L^−1^)	4.4 (0.6)	4.4 (0.5)	4.4 (0.5)	4.4 (0.5)	0.382
Urea (mmol·L^−1^)	9.2 (5.0)	8.5 (4.7)	7.9 (3.9)	6.7 (3.8)	**<0.0001**
Creatinine (u·moL^−1^)	125 (52)	121 (49)	116 (44)	107 (65)	**<0.0001**

**Table 3:** Unadjusted univariable predictors of all-cause mortality in patients with CHF (in order of Chi-square value).

Variables	*P* value	HR	95% CI		Chi-square
			Lower	Upper	
Log NT pro-BNP	<0.0001	1.63	1.53	1.74	230.8
6-MWT (m)*	<0.0001	0.968	0.964	0.973	184.1
Age (years)	<0.0001	1.05	1.04	1.06	137.2
Urea (mmol·L^−1^)	<0.0001	1.05	1.05	1.06	128.9
Haemoglobin (g·dL^−1^)	<0.001	0.84	0.81	0.87	96.9
NYHA class	<0.0001	1.64	1.48	1.82	89.0
Creatinine (u·moL^−1^)*	<0.001	1.023	1.016	1.029	58.3
Diastolic BP (mmHg)	<0.001	0.99	0.98	0.99	42.4
Sodium (mmol·L^−1^)	<0.001	0.96	0.94	0.97	27.1
QRS duration (ms)*	<0.001	1.05	1.03	1.07	18.3
BMI (kg·m^−2^)	<0.001	0.98	0.97	0.99	14.6
Systolic BP (mmHg)	<0.001	1.00	0.99	1.00	8.9
Heart rate (beats·min^−1^)	0.01	1.00	1.00	1.01	7.1

HR: hazard ratio; 95% CI: 95% confidence intervals; 6-MWT: 6-minute walk test; NYHA: New York Heart Association; LVI: left ventricular impairment; *HR reported for 10-unit increment.

**Table 4:** Bootstrap model based on 1000 stratified samples. Univariable predictors listed in order of magnitude of *P* value.

Variables	*B*	Bootstrap				
		Bias	SE	*P* value	95% CI	
					Lower	Upper
Log NT pro-BNP	0.489	−0.001	0.35	**0.001**	0.418	0.556
6-MWT (m)	−0.002	0.0001	0.0001	**0.001**	−0.002	−0.001
Age (years)	0.042	−0.001	0.004	**0.001**	0.033	0.050
NYHA class	0.136	0.004	0.060	**0.023**	0.027	0.263
Diastolic BP (mmHg)	−0.007	0.0001	0.003	**0.030**	−0.013	−0.001
Haemoglobin (g·dL^−1^)	−0.041	−0.002	0.021	**0.049**	−0.082	0.002
Urea (mmol·L^−1^)	0.024	−0.002	0.014	0.068	−0.006	0.047
Sodium (mmol·L^−1^)	−0.014	−0.001	0.010	0.182	−0.034	0.007
QRS duration (m·s^−1^)	0.001	0.0001	0.001	0.253	−0.001	0.004
Systolic BP (mmHg)	0.001	0.0001	0.002	0.528	−0.002	0.005
Creatinine (u·moL^−1^)	0.001	0.0001	0.001	0.667	−0.001	0.004
Heart rate (beats·min^−1^)	0.000	0.0001	0.002	0.736	−0.002	0.005
Potassium (mmol·L^−1^)	−0.001	0.0001	0.073	0.982	−0.143	0.137

SE: standard error; 95% CI: 95% confidence intervals; 6-MWT: 6-minute walk test; NYHA: New York Heart Association; LVI: left ventricular impairment.

**Table 5:** Multivariable predictors of long-term all-cause mortality in patients with CHF: final model (listed in order of magnitude of Wald statistic).

Variables	*P* value	Wald	HR	95% CI	
				Lower	Upper
Log NT pro-BNP	<0.0001	86.3	1.393	1.299	1.494
6-MWT (m)*	<0.0001	57.2	0.980	0.974	0.985
Age (years)	<0.0001	29.9	1.026	1.017	1.036
Diastolic BP (mmHg)	0.001	12.8	0.990	0.984	0.995
Urea (mmol·L^−1^)	0.002	9.5	0.980	0.974	0.985
Sodium (mmol·L^−1^)	0.052	3.8	0.978	0.957	1.000

HR: hazard ratio; 95% CI: 95% confidence intervals; 6-MWT: 6-minute walk test; NYHA: New York Heart Association; *HR reported for 10-unit increment.

## References

[1] Schalcher C, Rickli H, Brehm M (2003). Prolonged oxygen uptake kinetics during low-intensity exercise are related to poor prognosis in patients with mild-to-moderate congestive heart failure. *Chest*.

[2] Guazzi M, Adams V, Conraads V (2012). EACPR/AHA scientific statement. Clinical recommendations for cardiopulmonary exercise testing data assessment in specific patient populations. *Circulation*.

[3] Ingle L, Shelton RJ, Rigby AS, Nabb S, Clark AL, Cleland JGF (2005). The reproducibility and sensitivity of the 6-min walk test in elderly patients with chronic heart failure. *European Heart Journal*.

[4] Lipkin DP, Scriven AJ, Crake T, Poole-Wilson PA (1986). Six minute walking test for assessing exercise capacity in chronic heart failure. *British Medical Journal*.

[5] Olsson LG, Swedberg K, Clark AL, Witte KK, Cleland JGF (2005). Six minute corridor walk test as an outcome measure for the assessment of treatment in randomized, blinded intervention trials of chronic heart failure: a systematic review. *European Heart Journal*.

[6] Ingle L, Wilkinson M, Carroll S (2007). Cardiorespiratory requirements of the 6-min walk test in older patients with left ventricular systolic dysfunction and no major structural heart disease. *International Journal of Sports Medicine*.

[7] Crapo RO, Casaburi R, Coates AL (2002). ATS statement: guidelines for the six-minute walk test. *American Journal of Respiratory and Critical Care Medicine*.

[8] Bittner V, Weiner DH, Yusuf S (1993). Prediction of mortality and morbidity with a 6-minute walk test in patients with left ventricular dysfunction. *Journal of the American Medical Association*.

[9] Ingle L, Goode K, Rigby ASR, Cleland JGF, Clark AL (2006). Predicting peak oxygen uptake from 6-min walk test performance in male patients with left ventricular systolic dysfunction. *European Journal of Heart Failure*.

[10] Ingle L, Rigby AS, Carroll S (2007). Prognostic value of the 6 min walk test and self-perceived symptom severity in older patients with chronic heart failure. *European Heart Journal*.

[11] Guazzi M, Dickstein K, Vicenzi M, Arena R (2009). Six-minute walk test and cardiopulmonary exercise testing in patients with chronic heart failure: a comparative analysis on clinical and prognostic insights. *Circulation: Heart Failure*.

[12] National Institute for Clinical Excellence (NICE) (2003). *Chronic Heart Failure: Management of Chronic Heart Failure in Adults in Primary and Secondary Care*.

[13] Remme WJ, Swedberg K (2002). Comprehensive guidelines for the diagnosis and treatment of chronic heart failure: task force for the diagnosis and treatment of chronic heart failure of the European Society of Cardiology. *European Journal of Heart Failure*.

[14] Schiller NB, Shah PM, Crawford M (1989). Recommendations for quantitation of the left ventricle by two-dimensional echocardiography. American Society of Echocardiography Committee on Standards, Subcommittee on Quantitation of Two-Dimensional Echocardiograms. *Journal of the American Society of Echocardiography*.

[15] Bittner V, Weiner DH, Yusuf S (1993). Prediction of mortality and morbidity with a 6-minute walk test in patients with left ventricular dysfunction. *Journal of the American Medical Association*.

[16] Pocock SJ, Clayton TC, Altman DG (2002). Survival plots of time-to-event outcomes in clinical trials: good practice and pitfalls. *The Lancet*.

[17] Grambsch PM, Therneau TM (1994). Proportional hazards tests and diagnostics based on weighted residuals. *Biometrika*.

[18] Steyerberg EW, Eijkemans MJC, Harrell FE, Habbema JDF (2001). Prognostic modeling with logistic regression analysis: in search of a sensible strategy in small data sets. *Medical Decision Making*.

[19] Peduzzi P, Concato J, Feinstein AR, Holford TR (1995). Importance of events per independent variable in proportional hazards regression analysis II. Accuracy and precision of regression estimates. *Journal of Clinical Epidemiology*.

[20] Perneger TV (1998). What’s wrong with Bonferroni adjustments?. *British Medical Journal*.

[21] Manzano L, Babalis D, Roughton M (2011). Predictors of clinical outcomes in elderly patients with heart failure. *European Journal of Heart Failure*.

[22] Rostagno C, Olivo G, Comeglio M (2003). Prognostic value of 6-minute walk corridor test in patients with mild to moderate heart failure: comparison with other methods of functional evaluation. *European Journal of Heart Failure*.

[23] Beatty AL, Schiller NB, Whooley MA (2012). Six-minute walk test as a prognostic tool in stable coronary heart disease: data from the heart and soul study. *Archives of Internal Medicine*.

[24] Lucas C, Stevenson LW, Johnson W (1999). The 6-min walk and peak oxygen consumption in advanced heart failure: aerobic capacity and survival. *American Heart Journal*.

[25] Opasich C, Pinna GD, Mazza A (2001). Six-minute walking performance in patients with moderate-to-severe heart failure: is it a useful indicator in clinical practice?. *European Heart Journal*.

[26] Gitt AK, Wasserman K, Kilkowski C (2002). Exercise anaerobic threshold and ventilatory efficiency identify heart failure patients for high risk of early death. *Circulation*.

[27] Jankowska EA, Witkowski T, Ponikowska B (2007). Excessive ventilation during early phase of exercise: a new predictor of poor long-term outcome in patients with chronic heart failure. *European Journal of Heart Failure*.

[28] Arena R, Humphrey R, Peberdy MA (2003). Prognostic ability of VE/VCO_2_ slope calculations using different exercise test time intervals in subjects with heart failure. *European Journal of Cardiovascular Prevention and Rehabilitation*.

[29] Arena R, Myers J, Aslam SS, Varughese EB, Peberdy MA (2004). Influence of subject effort on the prognostic value of peak VO_2_ and the VE/VCO_2_ slope in patients with heart failure. *Journal of Cardiopulmonary Rehabilitation*.

[30] Arena R, Myers J, Abella J (2007). Development of a ventilatory classification system in patients with heart failure. *Circulation*.

[31] Guazzi M, Myers J, Peberdy MA, Bensimhon D, Chase P, Arena R (2008). Exercise oscillatory breathing in diastolic heart failure: prevalence and prognostic insights. *European Heart Journal*.

[32] Cohen-Solal A, Tabet JY, Logeart D, Bourgoin P, Tokmakova M, Dahan M (2002). A non-invasively determined surrogate of cardiac power (“circulatory power”) at peak exercise is a powerful prognostic factor in chronic heart failure. *European Heart Journal*.

[33] MacGowan GA, Janosko K, Cecchetti A, Murali S (1997). Exercise-related ventilatory abnormalities and survival in congestive heart failure. *The American Journal of Cardiology*.

[34] Scharf C, Merz T, Kiowski W, Oechslin E, Schalcher C, Brunner-La Rocca HP (2002). Noninvasive assessment of cardiac pumping capacity during exercise predicts prognosis in patients with congestive heart failure. *Chest*.

[35] Cicoira M, Davos CH, Francis DP (2004). Prediction of mortality in chronic heart failure from peak oxygen consumption adjusted for either body weight or lean tissue. *Journal of Cardiac Failure*.

[36] Chua TP, Ponikowski P, Harrington D (1997). Clinical correlates and prognostic significance of the ventilatory response to exercise in chronic heart failure. *Journal of the American College of Cardiology*.

[37] Francis DP, Shamim W, Davies LC (2000). Cardiopulmonary exercise testing for prognosis in chronic heart failure: continuous and independent prognostic value from VE/VCO_2_ slope and peak VO_2_. *European Heart Journal*.

[38] Ingle L, Witte KK, Cleland JGJF, Clark AL (2008). The prognostic value of cardiopulmonary exercise testing with a peak respiratory exchange ratio of < 1.0 in patients with chronic heart failure. *International Journal of Cardiology*.

[39] Davies LC, Francis DP, Piepoli M, Scott AC, Ponikowski P, Coats AJS (2000). Chronic heart failure in the elderly: value of cardiopulmonary exercise testing in risk stratification. *Heart*.

